# Optimisation of ANN topology for predicting the rehydrated apple cubes colour change using RSM and GA

**DOI:** 10.1007/s00521-016-2801-y

**Published:** 2016-12-24

**Authors:** Radosław Winiczenko, Krzysztof Górnicki, Agnieszka Kaleta, Monika Janaszek-Mańkowska

**Affiliations:** Faculty of Production Engineering, Warsaw University of Life Sciences, Nowoursynowska 164, 02-787 Warsaw, Poland

**Keywords:** Artificial neural networks, Genetic algorithms, Response surface methodology, Rehydration, Apple cubes

## Abstract

In this study, an efficient optimisation method by combining response surface methodology (RSM) and genetic algorithm (GA) is introduced to find the optimal topology of artificial neural networks (ANNs) for predicting colour changes in rehydrated apple cubes. A multi-layered feed-forward backpropagation ANN model of algorithms was developed to correlate one output (colour change) to four input variables (drying air temperature, drying air velocity, temperature of distilled water and rehydration time). A predictive model for ANN topology in terms of the best mean squared error (MSE) performance on validation samples was created using RSM. RSM model was integrated with an effective GA to find the optimum topology of ANN. The optimum ANN had minimum MSE when the number of hidden neurons, learning rate, momentum constant, number of epochs and number of training runs were 13, 0.33, 0.89, 3869 and 3, respectively. MSE of optimal ANN topology on validation samples was 0.0072095. It turned out that the optimal ANN topology can be considered as more precise for predicting colour change in the rehydrated apple cubes. Mean absolute error and regression coefficient (*R*) of the optimal ANN topology were determined as 0.0259 and 0.96475 for training, 0.0399 and 0.95243 for testing and 0.0264 and 0.95151 for validation data sets. The results of the testing model on new samples showed excellent agreement between the actual and predicted data with coefficient of determination *R* ^2^ = 0.97.

## Introduction

Rehydration is a complicated process aimed at the reinstatement, by way of the contact with water, of the features the dried material had prior to its pretreatment preceding the drying and the drying itself. The following three processes take place in the course of the rehydration: water absorption by the tissues of the dried material, thanks to which it increases its mass and volume as well as the leaching of water-soluble substances such as sugars, acids, vitamins and minerals from the rehydrated material [1, 2]. The progress of the discussed processes depends on the features of the raw material and conditions in which the drying with preceding pretreatments takes place [3]. Because of that, the progress of the rehydration process reflects the changes that took place in the raw material tissue as a result of the following processes: drying and pretreatment and also rehydration [4]. Such changes are the reason why the dried product fails to attain the features of the raw material after its rehydration, which shows irreversibility of the drying process [5].

Many studies of the rehydration process focused on the determination of rehydration indicators defining the reinstatement value of the dried material and on the process description using empirical formulae [6, 7]. There are also works oriented on the optimisation of parameters in selected technology of the thermal treatment [8, 9] and on the studying of changes in the tissue structure [10, 11], the chemical content [12] and colour [13, 14].

In the process of drying and rehydration, complex and highly nonlinear phenomena take place [15]. Therefore, it is difficult to estimate relationships between the input and the output of this complex system on the basis of mathematical approaches.

Intelligent techniques such as artificial neural networks show a high learning ability and capability of identifying mentioned systems [16]. The ability of neural networks to learn from repeated exposure to system characteristics has made them a popular choice for many applications, including drying technology [17, 18].

In the literature, several papers are related to modelling the heat and mass transfer kinetics [19], drying characteristics [20, 21], approximating the moisture content [22] and quality of apple tissue [23]. ANN models were also developed for predicting some physicochemical properties of apple tissue during hot air drying in thin layer [24]. Recently, several authors applied neural network for modelling heat and mass transfer during rehydration process [25, 26]. A simple ANN model was used to predict the shrinkage and estimate the rehydration capacity of the dried cooked rice [27] and dehydrated carrots [28]. A comprehensive review study devoted to application of ANNs in drying technology was made in paper [29].

Determination of the best ANN topology for estimation of colour change is usually put through by trial and error procedure that is very time-consuming [30]. Optimisation of neural networks parameters requests a large number of different topologies have to be constructed, trained and tested. However, there is no general rule used in selecting the value of variables in ANN. Also, it is dependent on the complexity of the system that is modelled.

In recent times, many researchers have used response surface methodology [31–33] and genetic algorithms [34, 35] to optimise ANN topology.

Genetic algorithm is a biologically inspired optimisation technique [36]. Recently, GA has gained popularity as a robust optimisation tool for multi-modal nonlinear problems. Sometimes GAs can exploit ANN models as their fitness function. In food industry, ANNs and GA system were used to control the fruit storage process. The authors [37, 38] indicated the need to apply a hybrid system for selection of input parameters (temperature, density) and output parameters (colour, mass loss, hardness) to improve the quality of the stored fruit. The authors noticed that the complex system of ANNs and GAs is superior to traditional computational techniques used in problems related to agriculture. ANN and GA system was successfully used for optimising thermal conditions for conduction-heated foods [39, 40]. Recently, ANN and GA approaches in drying technology have been described in papers [41–43].

It can be concluded from the literature review that the coupling of these two methods has many benefits for finding the global optimum neural networks topology and improving the model performance [33, 34].

The objective of this work is to optimise the neural network topology to predict the colour change in the rehydrated apple cubes using integrated RSM and GA methods.

## Materials and methods

### Material

High-quality Ligol apples were bought from the local market. They were washed in water, cut into cubes, with dimensions of 10 × 10 × 10 mm and were dried on the same day. The initial moisture content of a sample amounted to ca. 85% w.b. (5.66 d.b.).

### Drying equipments and experiments

The drying experiments were carried out in the dryer constructed in our laboratory. The details of dryer equipment and conducting the drying process can be found in a paper [44]. The laboratory dryer was run about 1 h, and when the steady conditions were achieved the samples were placed on a tray. The drying process lasted until the mass of the sample became constant. The drying experiments were performed at three levels of drying air temperatures of 50, 60 and 70 °C, together with two levels of air flow velocities—0.5 and 2 m/s. The final moisture content of dried apples amounted to ca. 9% w.b. (0.098 d.b.). Dry matter of the solid was determined according to AOAC standards [45]. The dried material obtained in the given conditions from three independent experiments was mixed and stored in a tightly sealed container for about one week at 20 °C; after that, samples were taken for further studies. The container in which the dried material was stored was placed in a cupboard, so the dried apples were not exposed to the sunlight.

### Rehydration procedure

The apple cubes were immersed in distilled water at four levels of temperatures—20, 45, 70 and 95 °C, using a water bath ELP 12 (LABOPLAY, Bytom, Poland). The initial mass of each dried sample subjected to rehydration was 10 g, and dried sample mass-to-medium mass ratio at the beginning of rehydration was 1:20. The rehydration lasted from 6 h (20 °C) to 2 h (95 °C). After removal from the water, the sample was dried on a filter paper. The medium was not stirred during the rehydration process, and its temperature was constant.

### Colour determination

**Fig. 1:**
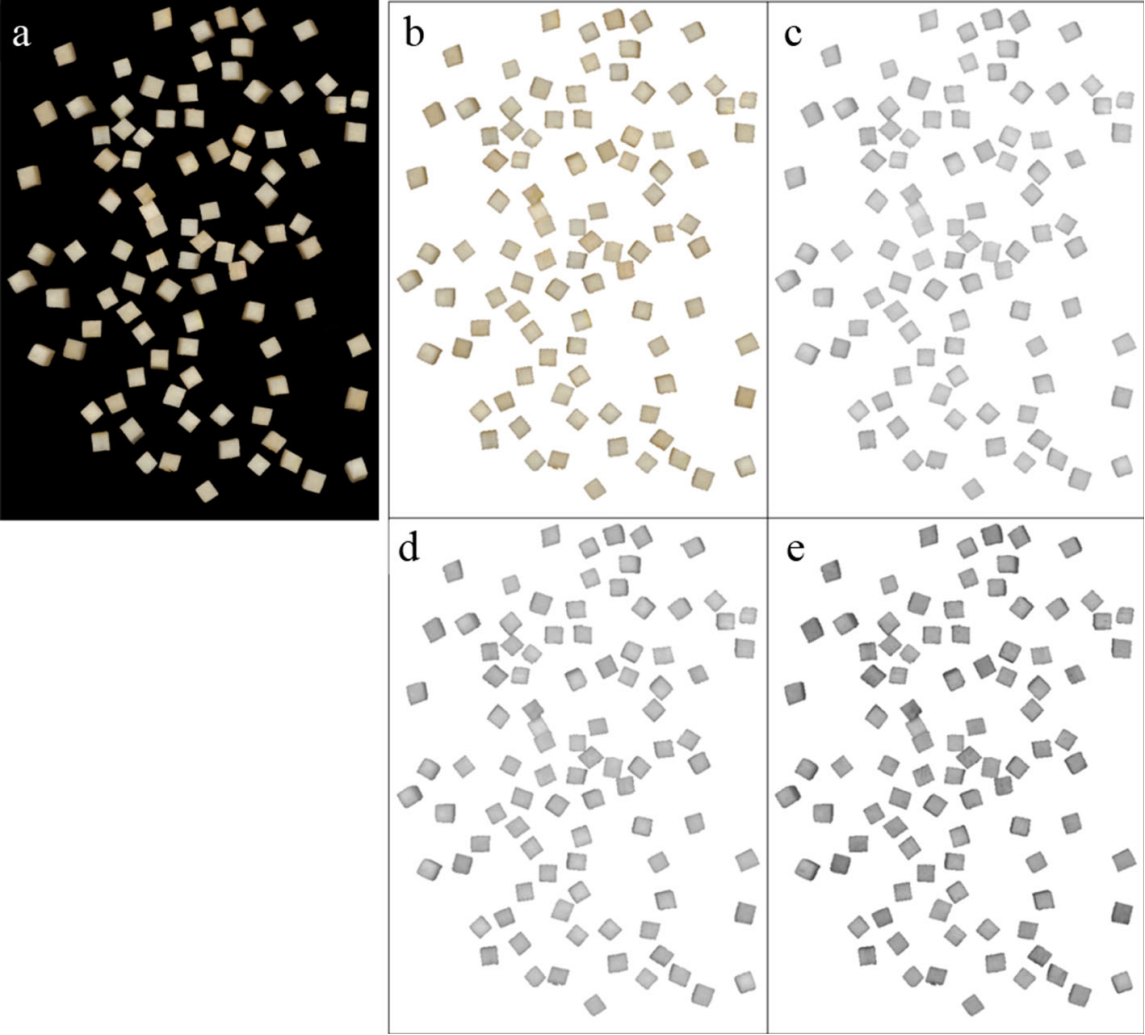
Original digital image of raw apple cubes—**a**, **b** preprocessed image extracted from the image background and **c** split into R, **d** split into G, **e** split into B channels

Colour images of fresh and rehydrated apple were acquired using a flatbed scanner (Canon CanoScan 5600F). The device was equipped with 6-line colour CCD sensor, fluorescent lamp and the 48-bit input/output interface (16 bits for each RGB channel). Images of resolution of 300 dpi were acquired to sRGB colour space and then saved in BMP format as matrixes with dimensions of 2552 × 3508 pixels. During the scanning process, all tools for an automatic image enhancement were disabled. Apple cubes were randomly positioned on the scanner platen. For fresh apple and each type of dehydrated cubes (various drying conditions: drying temperature, drying air velocity, and various rehydration temperatures and times) 30 images were acquired. The images were then transformed to CIEXYZ colour space [45, 46]. Nonlinear transformation of CIEXYZ to CIEL*a*b* coordinates was done relative to illuminant D50 and observer 10° according to CIE standard using 94.811, 100, 107.32 values as reference whites for X, Y and Z coordinates, respectively [47]. Chroma (C*) and hue (h*) of CIEL*C*h*colour space were calculated according to Schanda [48]. Original digital image of raw apple cubes and preprocessed image of apple cubes extracted from the image background is shown in Fig. 1.

## Quality of rehydrated product

It was also assumed that quality of the rehydrated product is defined by means of its colour change. The colour of a food product can be considered as a very important quality factor because it plays a decisive role in consumer’s acceptability. The colour change (*C*_ch_) was calculated according to the formula, given by [49]

1 $$C_{\text{ch}} = \sqrt {\left( {\frac{{\Delta L^{*} }}{{K_{L} S_{L} }}} \right)^{2} + \left( {\frac{{\Delta C^{*} }}{{K_{C} S_{C} }}} \right)^{2} + \left( {\frac{{\Delta H^{*} }}{{K_{H} S_{H} }}} \right)^{2} }$$

where *S*_*L*_ *, S*_*C*_ *, S*_*H*_ denote the weighing functions, adjusting the internal non-uniform structure of the CIEL^*^ *a* ^*^ *b* ^*^ and may be obtained using Eqs. (2–4)

2 $$S_{L} = 1$$

3 $$S_{C} = 1 + 0.045 \cdot C^{*}$$

4 $$S_{H} = 1 + 0.015 \cdot C^{*}$$

The parameters *K*_*L*_ *, K*_*C*_ *, K*_*H*_ express the variation from the reference conditions. The discussed parameters are equal to 1 in reference conditions [50]. Parameters Δ*L* ^*^, Δ*C* ^*^, Δ*H* ^*^ denote difference between the tested sample (_*T*_) and the standard (_*S*_) in terms of luminance, chroma and hue, respectively, and are determined according to formulae (5)–(7)

5 $$\Delta L^{*} = L_{T}^{*} - L_{S}^{*}$$

6 $$\Delta C^{*} = C_{T}^{*} - C_{S}^{*}$$

7 $$\Delta H^{*} = 2\sqrt {C_{T}^{*} \cdot C_{S}^{*} } \cdot \sin \left( {\frac{{\Delta h^{*} }}{2}} \right)$$

## Neural networks

### Design of ANN architecture

**Fig. 2:**
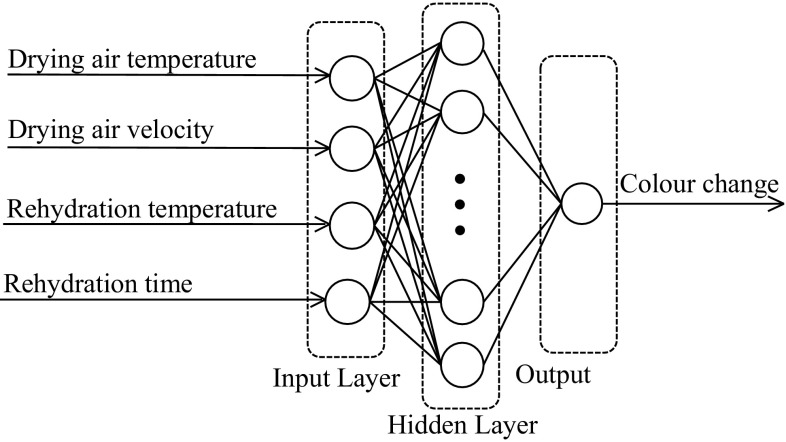
Schematic neural network architecture

A MLFF backpropagation (BP) neural model was developed (Fig. 2) for predicting the colour change in apple cubes during drying and rehydration processes. The following variables considered as the input parameters for the model were taken: drying air temperature, drying air velocity, temperature of distilled water, rehydration time. The network output variable included colour change. Since one output variable (colour change) was dependent on four exogenous input variables, one neuron was taken for the output whereas four neurons for the input layers. It was reported in earlier works [32, 33] that a network with one hidden layer and hyperbolic tangent sigmoid (tansig) function is commonly used for forecasting in practice [51, 52]. So, a single hidden layer network was used in this study. Therefore, for optimisation one hidden layer with tansig transfer function was considered. The tansig function was determined using Eq. (8)

8 $${\text{tansig}}\left( n \right) = \frac{2}{{1 + { \exp }\left( { - 2n} \right)}} - 1$$

Moreover, a linear (pureline) function for output was selected for simulation process.

### Data preprocessing

In order to produce the most efficient training, the data before training should be normalised. It is also helpful to analyse the network response after having completed the training. Therefore, to achieve this, 189 cases were chosen from our experiments. Chosen cases were randomly divided into the following sets: for training 133 samples (consisted of ≈70% cases), for validation 28 samples (≈15%) and for testing 28 samples (≈15%). The second data set was applied for evaluating the performance of the network in the process of training, while the third one was used for estimation of the predictive ability of the model which has been developed [51]. The data were normalised between 0.1 and 0.9 in the following way [53]

9 $$x_{{{\text{normalized}} \;{\text{value}} }} = 0.1 + 0.8\left( {\frac{{y_{{{\text{actual}}\;{\text{value}}}} - y_{{{\text{minimum}}\;{\text{value}}}} }}{{y_{{{\text{maximum}}\;{\text{value}}}} - y_{{{\text{minimum}}\;{\text{value}}}} }}} \right)$$

### Training methods

In the MLP networks, MSE can be obtained by various methods, including Levenberg–Marquardt (LM), gradient descent (GD) and conjugate gradient (CG). MLPs are as a rule trained using error backpropagation (BP) algorithm. It is a general method for iterative solution for weights and biases. BP uses GD technique. This technique is very slow at a small learning rate, but its convergence properties are slow. Different methods concentrated on speeding up BPs have been applied for an instance momentum term, variable learning rate. Finally, gradient descent momentum (GDM) algorithm has been chosen for training the networks. It avoids local minima, speeds up learning and stabilises convergence [33, 34]. Moreover, GDM allows a network to respond to the local gradient and to ignore small features in the error surface [51].

### Training parameters

In our training process, number of neurons in the hidden layer, number of epochs, learning rate and momentum coefficient are parameters that can affect the network simulation efficiency. However, GDM mainly depends on two training parameters: learning rate (*lr*) and momentum constant (*mc*). The first parameter determines the time indispensable for finding the minimum in the weight space. Too high *lr* leads to an increase in the magnitude of the oscillations for the MSE. Too small *lr* causes smaller steps taken in the weight space. Moreover, in this case the learning becomes slower and the capability of the network to escape from the local minima in the error surface becomes lower. The *mc* defines the amount of momentum. A *mc* of 1 result in a network that is totally insensitive to the local gradient. Consequently, *mc* does not learn properly. Too high *mc* causes diverging of the adaptation and gives unusable weights. Too small *mc* is responsible for a long learning time [32].

Number of neurons in the hidden layer is decisive for network performance. The small number of hidden neurons causes that the ANN is disable for adapting to a being modelled process, whereas when the number of hidden neurons is too big the system memorise errors [51]. Moreover, too many neurons do not propagate errors back efficiently [33] and therefore worsen the ability of the neural network to learn. Similar problems can be encountered while selecting the number of epochs. Too small number of epochs limits the ability of the network to process modelling. Too many epochs can lead to an overtraining of network and to an increasing of errors.

Therefore, figuring out the optimum values of affecting parameters for ANN is an important task and appropriate ranges should be chosen. The following numerical variables were chosen for ANN optimisation number of neurons in the hidden layer, *lr*, *mc*, epoch number and number of training runs. The responses sought were faulty on the best validation performance. BP uses a GD technique. Its stability depends on *lr*. Small *lr* leads to very stable GD. In the MATLAB 7.0 software, the defaults value for *lr* and *mc* equal 0.01 and 0.9, respectively. Accordingly, we changed *lr* from 0.01 to 0.4 and *mc* between 0.1 and 0.9. Similarly, the number of neurons in the hidden layer (2–16), training epoch (300–5000) and number of training runs (3–7) were chosen. The range of input variables in ANN model is shown in Table 1.

**Table 1:** Limit of input variables in the neural network model

Parameters	Down	Up
*x*_1_: Neurons number	2	16
*x*_2_: Learning rate	0.01	0.4
*x*_3_: Training epoch	300	5000
*x*_4_: Momentum constant	0.1	0.9
*x*_5_: Number of training runs	3	7

### Performance evaluation

After having found the optimal ANN topology, measuring its performance is the next step. The performance of the designed ANN was estimated on the basis of coefficient of determination (*R* ^2^), mean square error (MSE) and mean absolute error (MAE) [33]. Discussed parameters were determined using Eqs. (10–12)

10 $$R^{2} = 1 - \frac{{\mathop \sum \nolimits_{i = 1}^{n} \left( {x_{pi} - x_{di} } \right)^{2} }}{{\mathop \sum \nolimits_{i = 1}^{n} \left( {x_{pi} - \bar{x}} \right)^{2} }}$$

11 $${\text{MSE}} = \frac{1}{N}\mathop \sum \limits_{i = 1}^{n} \left( {x_{pi} - x_{di} } \right)^{2}$$

12 $${\text{MAE}} = \frac{1}{N}\mathop \sum \limits_{i = 1}^{n} \left| {\left( {x_{pi} - x_{di} } \right)} \right|$$

where *x*_*pi*_ is the network (predicted) output derived from observation *i*, *x*_*di*_ is an experimental (actual) output derived from observation *i*, $\bar{x}$ is the average value of an experimental output, and *N* is the number of data. MSE informs of the differences between the value implied by the estimator and estimated quantity. The value of MSE close to 0 indicates that the network can be considered as a satisfactory one. *R* ^2^ informs of the correctness of model fitting. If *R* ^2^ = 1, the regression line fits the data excellent. MAE shows how close the predictions are to the final outcomes. The value of MAE close to 0 indicates that the error of our model decreases.

## Hybrid intelligent system

**Fig. 3:**
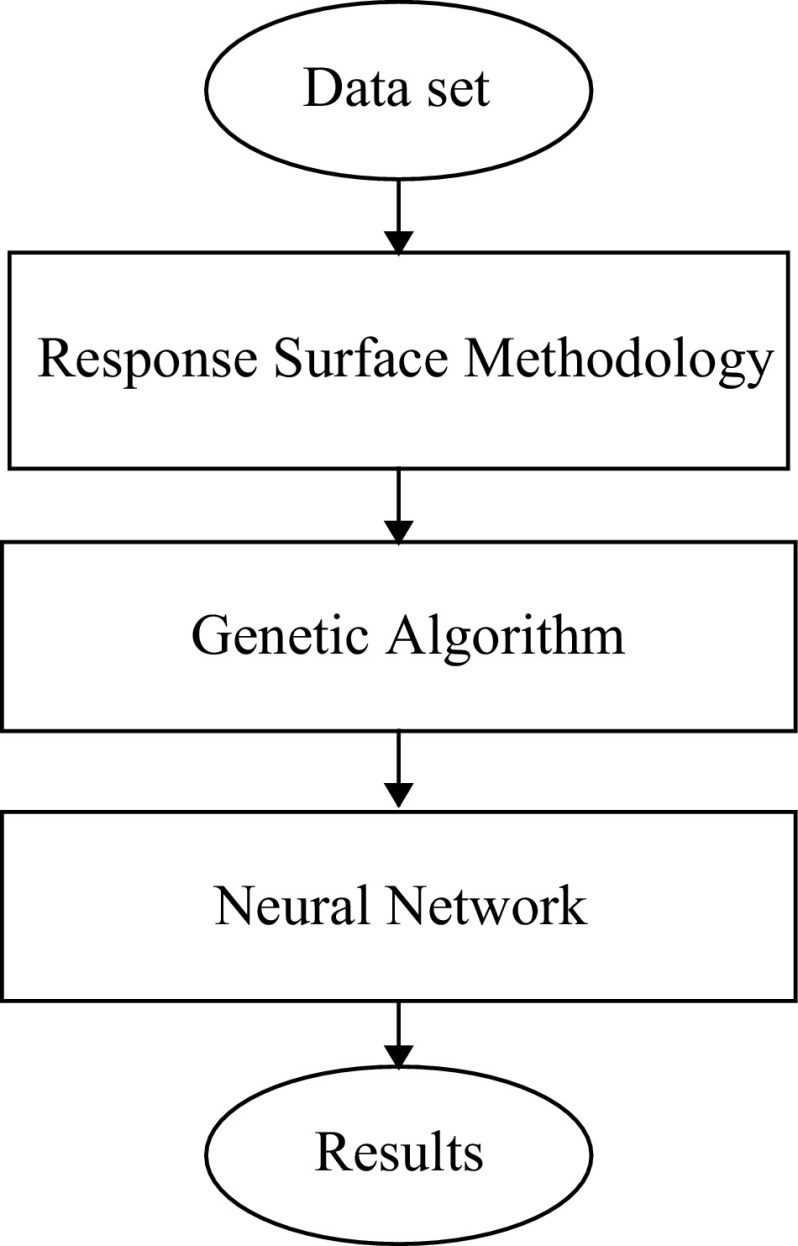
Block diagram of simulation process

Figure 3 provides a schematic diagram of simulation system. The proposed hybrid RSM–ANN–GA system is described briefly below. This system includes the following steps:

- *Step 1* Collection of the data set
- *Step 2* RSM designs the experiment and builds a fitness function
- *Step 3* GA optimises ANN architecture
- *Step 4* ANN predicts the colour change

The algorithm proceeded with its iterations until a specified performance criterion became satisfactory. More details related to applied RSM and GA algorithm are described in Sects. 5.1 and 5.2.

### Response surface method

The optimum architecture of ANN was determined on the basis of the runs designed by response surface methodology. A face-centred full central composite design of five numerical factors (number of neurons in the hidden layer, learning rate, momentum constant, training epochs and number of training times) with three-level factorial design matrix was selected. The experimental design matrix (see Table 2) consisting of 50 set of conditions and comprising a full replication of five-factor factorial design of 32 points, 10 star points and 8 centre points was used. The upper and lower limits of the parameters were coded as +1 and −1, respectively.

**Table 2:** Mean square error (MSE) results obtained by various neural network configuration

Design points	Experiment ID	Number of neurons	Learning rate	Momentum constant	Training epoch	Number of training run	MSE
Points	1	2	0.01	0.1	300	3	0.3518
	2	2	0.01	0.1	300	7	0.0827
	3	2	0.01	0.1	5000	3	0.1879
	4	2	0.01	0.1	5000	7	0.0955
	5	2	0.01	0.9	300	3	0.3472
	6	2	0.01	0.9	300	7	0.1361
	7	2	0.01	0.9	5000	3	0.1933
	8	2	0.01	0.9	5000	7	0.1848
	9	2	0.40	0.1	300	3	0.1358
	10	2	0.40	0.1	300	7	0.0322
	11	2	0.40	0.1	5000	3	0.0240
	12	2	0.40	0.1	5000	7	0.0161
	13	2	0.40	0.9	300	3	0.1089
	14	2	0.40	0.9	300	7	0.0470
	15	2	0.40	0.9	5000	3	0.0308
	16	2	0.40	0.9	5000	7	0.0200
	17	16	0.01	0.1	300	3	0.1953
	18	16	0.01	0.1	300	7	0.0868
	19	16	0.01	0.1	5000	3	0.0217
	20	16	0.01	0.1	5000	7	0.0174
	21	16	0.01	0.9	300	3	0.1330
	22	16	0.01	0.9	300	7	0.0647
	23	16	0.01	0.9	5000	3	0.0237
	24	16	0.01	0.9	5000	7	0.0330
	25	16	0.40	0.1	300	3	0.0306
	26	16	0.40	0.1	300	7	0.0169
	27	16	0.40	0.1	5000	3	0.0074
	28	16	0.40	0.1	5000	7	0.0085
	29	16	0.40	0.9	300	3	0.0174
	30	16	0.40	0.9	300	7	0.0116
	31	16	0.40	0.9	5000	3	0.0080
	32	16	0.40	0.9	5000	7	0.0086
Star points	33	2	0.205	0.5	2650	5	0.0305
	34	9	0.010	0.5	2650	5	0.0273
	35	9	0.205	0.1	2650	5	0.0092
	36	9	0.205	0.5	300	5	0.0576
	37	9	0.205	0.5	2650	3	0.0084
	38	9	0.205	0.5	2650	7	0.0102
	39	9	0.205	0.5	5000	5	0.0110
	40	9	0.205	0.9	2650	5	0.0105
	41	9	0.40	0.5	2650	5	0.0108
	42	16	0.205	0.5	2650	5	0.0089
Centre points	43	9	0.205	0.5	2650	5	0.0155
	44	9	0.205	0.5	2650	5	0.0094
	45	9	0.205	0.5	2650	5	0.0108
	46	9	0.205	0.5	2650	5	0.0149
	47	9	0.205	0.5	2650	5	0.0108
	48	9	0.205	0.5	2650	5	0.0127
	49	9	0.205	0.5	2650	5	0.0098
	50	9	0.205	0.5	2650	5	0.0104

Fifty different patterns of proposed ANNs (Table 2) were designed and trained to model the best MSE performance on validation data set using the Design-Expert (DOE) software. Finding a suitable approximation for the true efficient relationship between the response and the set of independent variables makes the first stage in discussed methodology [54, 55]. The response variables were then transformed to natural logarithm function. It makes the distribution of the response variable closer to the normal distribution and improves the model fitting to the data. The experimental results of the CCD were fitted with a second-order polynomial equation using a multiple regression technique. Equation (13) represents the quadratic model for predicting the optimal point

13 $$Y = \beta_{0} + \mathop \sum \limits_{i = 1}^{k} \beta_{i} x_{i} + \mathop \sum \limits_{i = 1}^{k} \beta_{ii} x_{i} x_{i} + \mathop \sum \limits_{i = 1}^{k - 1} \mathop \sum \limits_{j = i + 1}^{k} \beta_{ij} x_{i} x_{j} + \varepsilon_{ij}$$

where *Y* is the response (MSE), $\beta_{0} , \beta_{i} , \beta_{ii} ,$ and *β*_*ij*_ are regression coefficients (intercept, linear, quadratic and interaction, respectively), *x*_i_ and *x*_j_ are the independent variables, *k* is the number of levels, and *ɛ*_*ij*_ is an error observed in the response.

### Genetic algorithm

GAs use the evolutionary principle of survival characteristics for the best adapted chromosomes [56]. A group of chromosomes is called a population. Each population of chromosomes has the same size which is referred to as population size. According to the researchers [32, 33, 57], a suitable population size numbers about 20–30 chromosomes. However, sometimes a population size with 50–80 has lead to the best answers [58, 59]. With a large population size, the GA searches the solution space more thoroughly, thereby reducing the chance that the algorithm returns a local minimum that is not a global minimum. In addition, a large population size causes the algorithm to run more slowly [58, 60].

The main data structures in GA toolbox are chromosomes, objective function values and fitness values. The chromosome data structure stores an entire population in a single matrix of size Nind × Lind, where Nind is the number of individuals in the population and Lind is the length of the genotypic representation of these individuals. Each of the rows correspond to an individual’s genotype, consisting of base-n, typically binary, values

$${\text{Chrome}} = \left[ {\begin{array}{*{20}c} {{\text{g}}_{1.1} } & {{\text{g}}_{1.2} } & {{\text{g}}_{1.3} } & \ldots & {{\text{g}}_{{1.{\text{Lind}}}} } \\ {{\text{g}}_{2.1} } & {{\text{g}}_{2.2} } & {{\text{g}}_{2.3} } & \ldots & {{\text{g}}_{{2.{\text{Lind}}}} } \\ {{\text{g}}_{3.1} } & {{\text{g}}_{3.2} } & {{\text{g}}_{3.3} } & \ldots & {{\text{g}}_{{3.{\text{Lind}}}} } \\ .& .& .& \ldots & .\\ {{\text{g}}_{Nind.1} } & {{\text{g}}_{{{\text{Nind}}.2}} } & {{\text{g}}_{{{\text{Nind}}.3}} } & \ldots & {{\text{g}}_{{{\text{Nind}}.{\text{Lind}}}} } \\ \end{array} } \right]\begin{array}{*{20}c} {{\text{individual}}\;1} \\ {{\text{individual}}\;2} \\ {{\text{individual}}\;3} \\ . \\ {{\text{individual}}\;{\text{Nind}}} \\ \end{array}$$

Such a data representation does not force the chromosome structure, requiring only all chromosomes to be of equal length. Thus, structured populations or populations with varying genotypic bases may be used in the GA toolbox provided that a suitable decoding function, mapping chromosomes onto phenotypes, is employed.

The decision variables (phenotypes) in the GA are obtained by applying some mapping from the chromosome representation into the decision variable space. Here, each of the strings contained in the chromosome structure decodes to a row vector of order Nvar, according to the number of dimensions in the search space and corresponding to the decision variable vector value. The decision variables are stored in a numerical matrix of size Nind × Nvar. Again, each of the rows corresponds to a particular individual’s phenotype. An example of the phenotype data structure is given below, where bin2real is used for representation of an arbitrary function, possibly from the GA Toolbox, mapping the genotypes onto the phenotypes.

$$\begin{aligned} {\text{Phen }} & = {\text{ bin2real}}\left( {\text{Chrom}} \right)\;\% \;{\text{map}}\;{\text{genotype}}\;{\text{to}}\;{\text{phenotype}} \\ {\text{Phen}} & = \left[ {\begin{array}{*{20}c} {{\text{x}}_{1.1} } & {{\text{x}}_{1.2} } & {{\text{x}}_{1.3} } & \ldots & {{\text{x}}_{{1.{\text{Lind}}}} } \\ {{\text{x}}_{2.1} } & {{\text{x}}_{2.2} } & {{\text{x}}_{2.3} } & \ldots & {{\text{x}}_{{2.{\text{Lind}}}} } \\ {{\text{x}}_{3.1} } & {{\text{x}}_{3.2} } & {{\text{x}}_{3.3} } & \ldots & {{\text{x}}_{{3.{\text{Lind}}}} } \\ .& .& .& \ldots & .\\ {{\text{x}}_{{{\text{Nind}}.1}} } & {{\text{x}}_{{{\text{Nind}}.2}} } & {{\text{x}}_{{{\text{Nind}}.3}} } & \ldots & {{\text{x}}_{{{\text{Nind}}.{\text{Lind}}}} } \\ \end{array} } \right]\;\begin{array}{*{20}c} {{\text{individual}}\;1} \\ {{\text{individual}}\;2} \\ {{\text{individual}}\;3} \\ . \\ {{\text{individual}}\;{\text{Nind}}} \\ \end{array} \\ \end{aligned}$$

An objective function is used for evaluation of the performance of the phenotypes in the problem domain. Objective function values can be scalar or, in the case of multi objective problems, vectorial. Discussed values are not necessarily the same as the fitness values. Objective function values are stored in a numerical matrix of size Nind × Nobj, where Nobj is the number of objectives. Each of the rows corresponds to a particular individual’s objective vector.

$$\begin{aligned} {\text{Objv}} & = {\text{ OBJFUN}}\left( {\text{Phen}} \right)\;\% \;{\text{Objective}}\;{\text{Function}} \\ {\text{Objv}} & = \left[ {\begin{array}{*{20}c} {{\text{y}}_{1.1} } & {{\text{y}}_{1.2} } & {{\text{y}}_{1.3} } & \ldots & {{\text{y}}_{{1.{\text{Lind}}}} } \\ {{\text{y}}_{2.1} } & {{\text{y}}_{2.2} } & {{\text{y}}_{2.3} } & \ldots & {{\text{y}}_{{2.{\text{Lind}}}} } \\ {{\text{y}}_{3.1} } & {{\text{y}}_{3.2} } & {{\text{y}}_{3.3} } & \ldots & {{\text{y}}_{{3.{\text{Lind}}}} } \\ .& .& .& \ldots & .\\ {{\text{y}}_{{{\text{Nind}}.1}} } & {{\text{y}}_{{{\text{Nind}}.2}} } & {{\text{y}}_{{{\text{Nind}}.3}} } & \ldots & {{\text{y}}_{{{\text{Nind}}.{\text{Lind}}}} } \\ \end{array} } \right]\begin{array}{*{20}c} {{\text{individual}}\;1} \\ {{\text{individual}}\;2} \\ {{\text{individual}}\;3} \\ . \\ {{\text{individual}}\;{\text{Nind}}} \\ \end{array} \\ \end{aligned}$$

Fitness values are derived from the objective function values through a scaling or ranking function. Fitnesses are non-negative scalars and are stored in the column vectors of length Nind, an example of which is shown below. Again, ranking is an arbitrary fitness function [61].

$${\text{Fitn}} = {\text{ranking}}\left( {\text{ObjV}} \right).$$

$$\begin{array}{llll} {\text{Fitn}} =& {f_{1} } &{{\text{individual}}\;1} \\ &{f_{2} } & {{\text{individual}}\;2} \\ &{f_{3} } & {{\text{individual}}\;3} \\ &\cdots & {} \\ &{f_{\text{Nind}} } & {{\text{individual}}\;{\text{Nind}}} \\ \end{array}$$

**Fig. 4:**
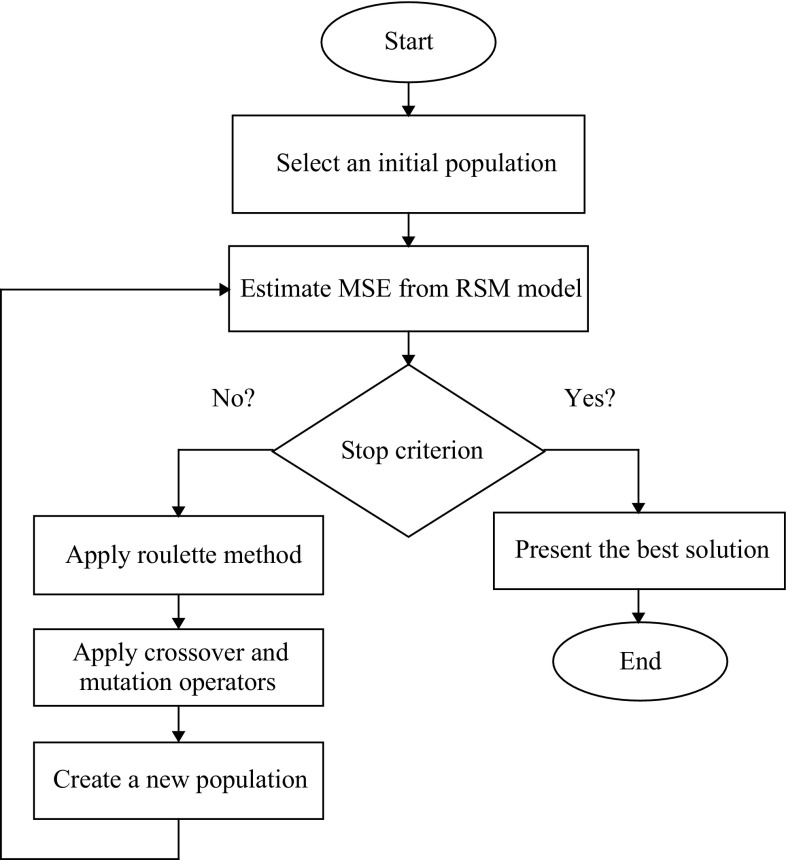
General structure of the genetic algorithm

The general steps of a genetic algorithm are presented in Fig. 4. This algorithm encodes a possible solution to a particular problem on a simple chromosome string and applies specified operators to a chromosome for preserving critical information and producing a new set of population with the aim to generate strings which map to high function values [36]. The main GA operators are selection, crossover and mutation (see Table 3). Roulette as selection method was used in the study. Roulette simulates a roulette wheel with the area of each segment proportional to its expectation. GA then uses a random number to select one of the sections with a probability equal to its area. The next main operator is crossover. Crossover combines two chromosomes, or child, for the next generation. A single point as a crossover function was applied in the study. Single point chooses a random integer *n* between 1 and a number of variables, selects the vector entries numbered less than or equal to *n* from the first parent, selects genes numbered greater than *n* from the second parent, and concatenates these entries to form the child.

**Table 3:** Genetic algorithm operators

Population size	30
Selection method	Roulette
Crossover rate	0.95
Crossover function	Single point
Mutation rate	0.15
Mutation function	Uniform
Number generations	3000
Stall generations	2000

For example, if *p*1 and *p*2 are the parents

$$\begin{aligned} p1 \, & = \, \left[ {{\text{a}}\;{\text{b}}\;{\text{c}}\;{\text{d}}\;{\text{e}}\;{\text{f}}\;{\text{g}}\;{\text{h}}} \right] \\ p2 \, & = \, \left[ {1 \, 2 \, 3 \, 4 \, 5 \, 6 \, 7 \, 8} \right] \\ \end{aligned}$$

and the crossover point is 3, the function returns the following child

$${\text{child}} = \left[ {{\text{a}}\;{\text{b}}\;{\text{c}}\;4\;5\;6\;7\;8} \right].$$

The next parameter of GA is mutation. This operator makes small random changes in the individuals of the population, which provide genetic diversity and enable GA to search a broader space. Uniform as the mutation function was applied in the simulation process. In this case, GA selects a fraction of the vector entries of chromosomes for mutation, where each entry has the same probability as the mutation rate of being mutated. Next, the algorithm replaces each selected entry by a random number selected uniformly from the range of that entry.

The simulation process proceeds by 58 s. The computer simulations were conducted using the computer with the following specifications: Intel Core i5-2400s, processor 2.50 GHz speed, 6 GB memory and commercially available ANN software, MATLAB 7.0 [56].

## Results

### Statistical test results

The MSE results on validation data set are given in Table 2. In this study, the cubic model (CM) was chosen to give the correlation between neural network effective factors and the response of ln(MSE). Moreover, mentioned model was selected due to high amount of *R* and non-significant lack of fit. Finally, the selected CM was reduced to modified cubic model (MCM) on the basis of low *P* value and high ln(MSE) values. The results of the reduced CM in the form of ANOVA are shown in Table 4. ANOVA gives a very high *F* value (121.20) and a very low *P* value (<0.0001). The model implies the model significant. The prediction *R* ^2^ (0.973) is very close to adjusted *R* ^2^ (0.982). The difference is <0.2. Moreover, adequate precision that measures the signal-to-noise ratio (37.359) indicates an adequate signal.

**Table 4:** ANOVA for predicted RSM model

Source	Sum of squares	DOF	Mean square	*F* value	*P* value
Model	64.67	23	2.81	121.20	<0.0001
*x*_1_: Number of neurons	12.72	1	12.72	548.38	<0.0001
*x*_2_: Learning rate	0.43	1	0.43	18.53	0.0002
*x*_3_: Momentum constant	0.02	1	0.02	0.95	0.3397
*x*_4_: Training epoch	1.37	1	1.37	59.08	<0.0001
*x*_5_: Number of training run	0.02	1	0.02	0.81	0.3757
*x*_1_ *x*_2_	0.00	1	0.00	0.11	0.7425
*x*_1_ *x*_3_	0.26	1	0.26	11.04	0.0027
*x*_1_ *x*_4_	0.59	1	0.59	25.41	<0.0001
*x*_1_ *x*_5_	0.44	1	0.44	18.87	0.0002
*x*_2_ *x*_3_	0.09	1	0.09	3.97	0.0571
*x*_2_ *x*_4_	0.03	1	0.03	1.42	0.2446
*x*_3_ *x*_4_	0.23	1	0.23	9.71	0.0044
*x*_3_ *x*_5_	0.15	1	0.15	6.57	0.0165
*x*_4_ *x*_5_	1.01	1	1.01	43.74	<0.0001
*x*_1_^2^	0.36	1	0.36	15.72	0.0005
*x*_2_^2^	0.45	1	0.45	19.40	0.0002
*x*_4_^2^	1.66	1	1.66	71.73	<0.0001
*x*_5_^2^	0.11	1	0.11	4.68	0.0400
*x*_1_ *x*_2_ *x*_4_	1.33	1	1.33	57.53	<0.0001
*x*_1_ *x*_3_ *x*_4_	0.09	1	0.09	3.71	0.0651
$x_{2}^{2}$ *x*_2_	0.15	1	0.15	6.29	0.0187
$x_{2 }^{2}$ *x*_4_	0.24	1	0.24	10.54	0.0032
$x_{2}^{2}$ *x*_5_	0.26	1	0.26	11.03	0.0027
Residual	0.60	26	0.02		
Lack of fit	0.35	19	0.02	0.52	0.8772
Pure error	0.25	7	0.04		
Cor total	65.28	49			

The results of statistical test show that the first-order effect of neurons number was the most significant term in estimation of ln(MSE). It was followed by a training epoch and *lr*, respectively, whereas *mc* and number of training runs had no significant effect on the responses. Similar results in case of *lr* and training epoch were reported by [33, 34].

### Mathematical model results

Finally, a MCM term of coded value is:

14 $$\begin{aligned} { \ln }\left( {\text{MSE}} \right) & = - 4.48 - 0.611 \cdot x_{1} - 0.463 \cdot x_{2} + 0.025 \cdot x_{3} - 0.82 \cdot x_{4} + 0.097 \cdot x_{5} \\ \quad + 0.008 \cdot x_{1} \cdot x_{2} - 0.08 \cdot x_{2} \cdot x_{3} - 0.13 \cdot x_{1} \cdot x_{4} + 0.11 \cdot x_{1} \cdot x_{5} - 0.05 \cdot x_{2} \cdot x_{3} - 0.032 \cdot x_{2} \cdot x_{4} \\ \quad + 0.08 \cdot x_{3} \cdot x_{4} + 0.069 \cdot x_{3} \cdot x_{5} + 0.17 \cdot x_{4} \cdot x_{5} + 0.37 \cdot x_{1}^{2} + 0.41 \cdot x_{2}^{2} + 0.79 \cdot x_{4}^{2} - 0.20 \cdot x_{5}^{2} \\ \quad + 0.20 \cdot x_{1} \cdot x_{2} \cdot x_{4} + 0.051 \cdot x_{1} \cdot x_{3} \cdot x_{4} - 0.27 \cdot x_{1}^{2} \cdot x_{2} + 0.36 \cdot x_{1}^{2} \cdot x_{4} - 0.36 \cdot x_{1}^{2} \cdot x_{5} \\ \end{aligned}$$

where $x_{1} , x_{2} , x_{3} , x_{4, }$ and *x*_5_ parameters are defined in Table 1. This model was checked hierarchically. The above statistical estimators indicate an adequate neural model with optimal structure that can be used for prediction of colour change in rehydrated apple cubes.

### RSM and contour plot results

**Fig. 5:**
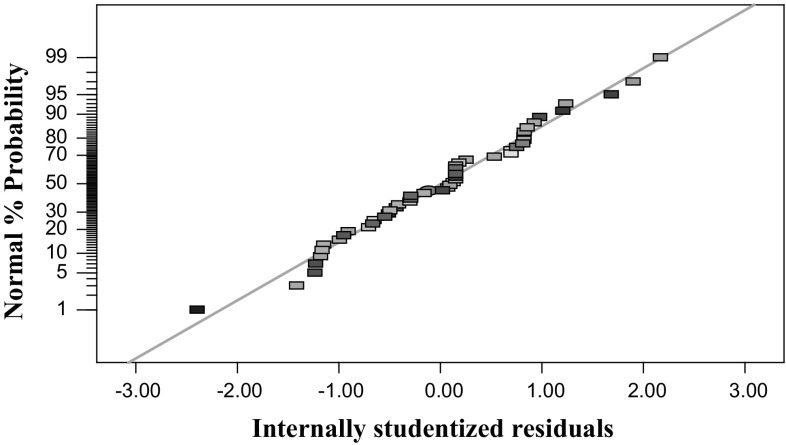
Normal probability of the internally studentised residuals

**Fig. 6:**
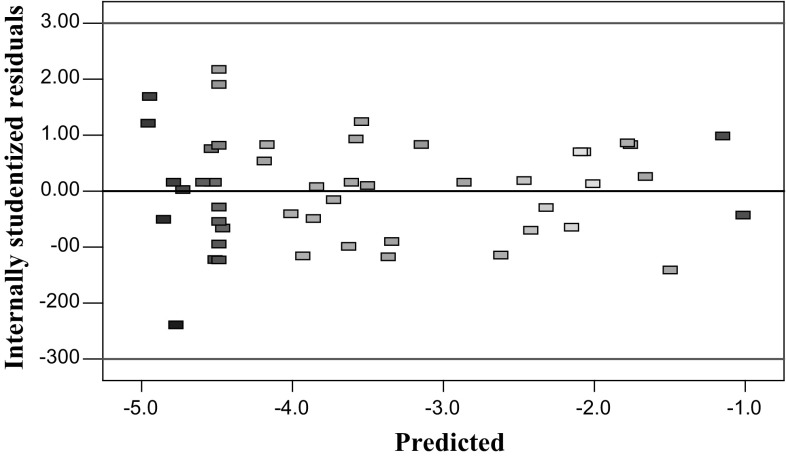
Internally studentised residuals versus predicted response

Figure 5 shows the effect of normal percent probability on the internally studentised residuals. As can be seen from a residual plot, the linear function very well approximates the results. Moreover, the residual scatters randomly on the display (Fig. 6), suggesting that the variance of the original observation is constant for all responses. Therefore, it can be concluded from these plots that the empirical model is suitable for describing relationships between design variables described by RSM.

**Fig. 7:**
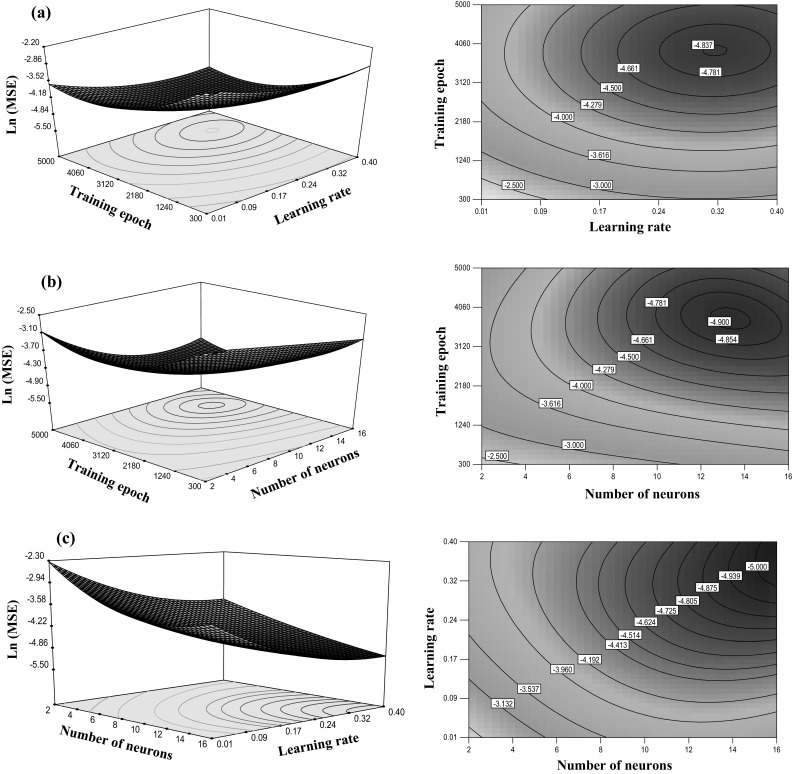
Response surface and contour plots of ln(MSE) for: **a** training epoch and learning rate, **b** training epoch and number of neurons in the hidden layer and **c** learning rate and number of neurons in the hidden layer

Figure 7 shows the response surfaces (RSs) and contour plots (CPs) obtained by Design-Expert (DOE) software. Each graph represents a combination of two factors at the time and holding all other factors at the middle level. The effect of different values of neurons number and training epoch on ln(MSE) can be predicted from the RSs and CPs as shown in Fig. 7a, b. It is obvious that minimum value of MSE can be found by 3000–4000 epochs and 0.25–0.35 *lr*. Moreover, this range was observed for epoch number in relation with number of neurons (Fig. 7b). The CPs show that along with an increase in number of neurons from 2 to 16 and *lr* from 0.01 to 0.4, the ln(MSE) decreases to −5 (see Fig. 7c).

**Fig. 8:**
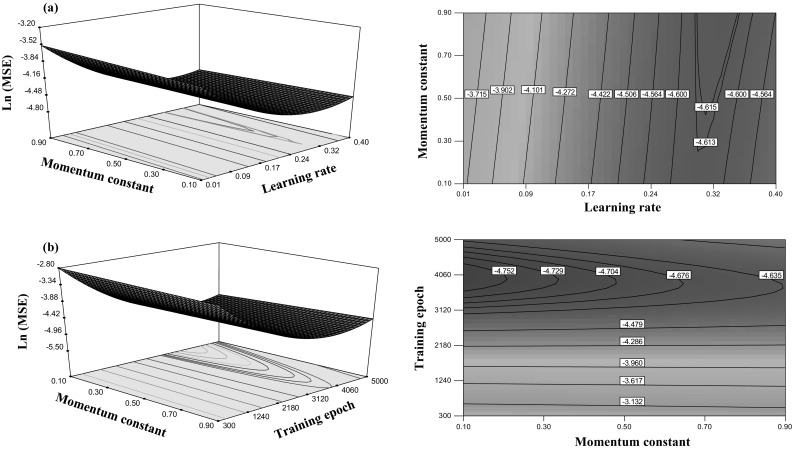
Response surface and contour plots of ln(MSE) for: **a** learning rate and momentum constant and **b** training epoch and momentum constant

The response surface plot of momentum constant and learning rate is shown in Fig. 8a. The CP shows that along with an increase in *lr* from 0.01 to 0.4 and *mc* from 0.1 to 0.5, the ln(MSE) decreases to −4.615. Figure 8b shows the contour plot, where along with an increase in training epochs from 3000 to 4500 and *mc* to 0.5, the ln(MSE) decreases to −4.75.

### GA optimisation results

The fitness function Eq. (14) developed by the RSM model was applied for predicting the colour change in rehydrated apple cubes. The fitness function was a function minimising the ln(MSE) in experimental ranges presented in Table 1.

**Fig. 9:**
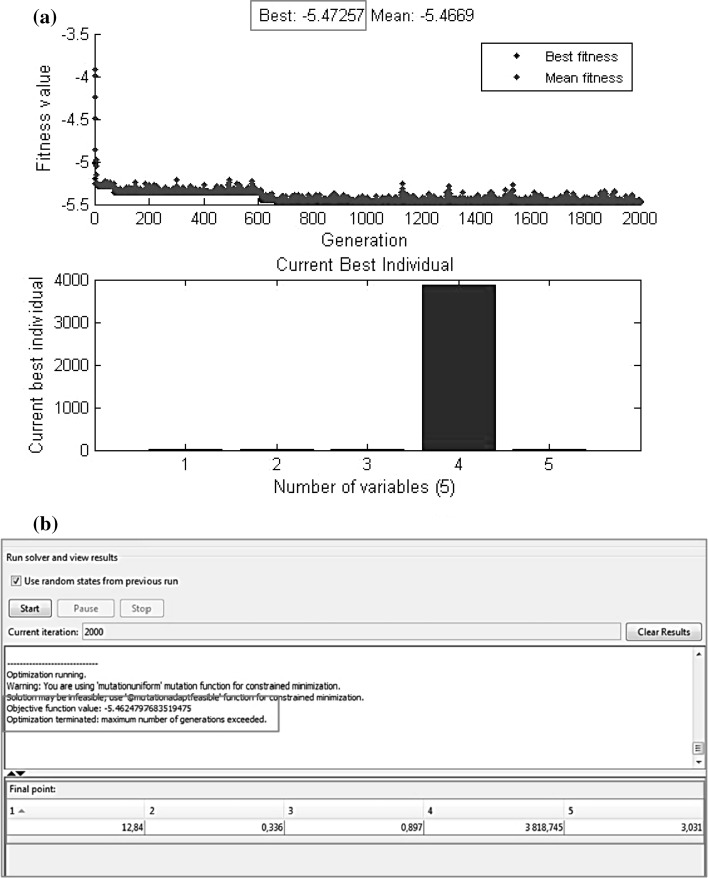
Results of genetic algorithm optimisation:** a**) converged values of drying and rehydration parameters,** b**) convergence of fitness values

As can be noticed in Fig. 9a, the optimisation terminated when maximum number of generations exceeded 2000 iterations. The objective function value ln(MSE) = −5.47257 was obtained for the final points presented in Fig. 9b. Table 5 shows results of optimised ANN parameters. The optimum values were as follows: number of neurons = 13, training epoch = 3869, *lr* = 0.33, *mc* = 0.89 and number of training runs = 3.

**Table 5:** Optimised ANN parameters

Parameters					MSE
*x*_1_	*x*_2_	*x*_3_	*x*_4_	*x*_5_	
13	0.33	0.89	3869	3	0.0072095

### Errors of model results

**Fig. 10:**
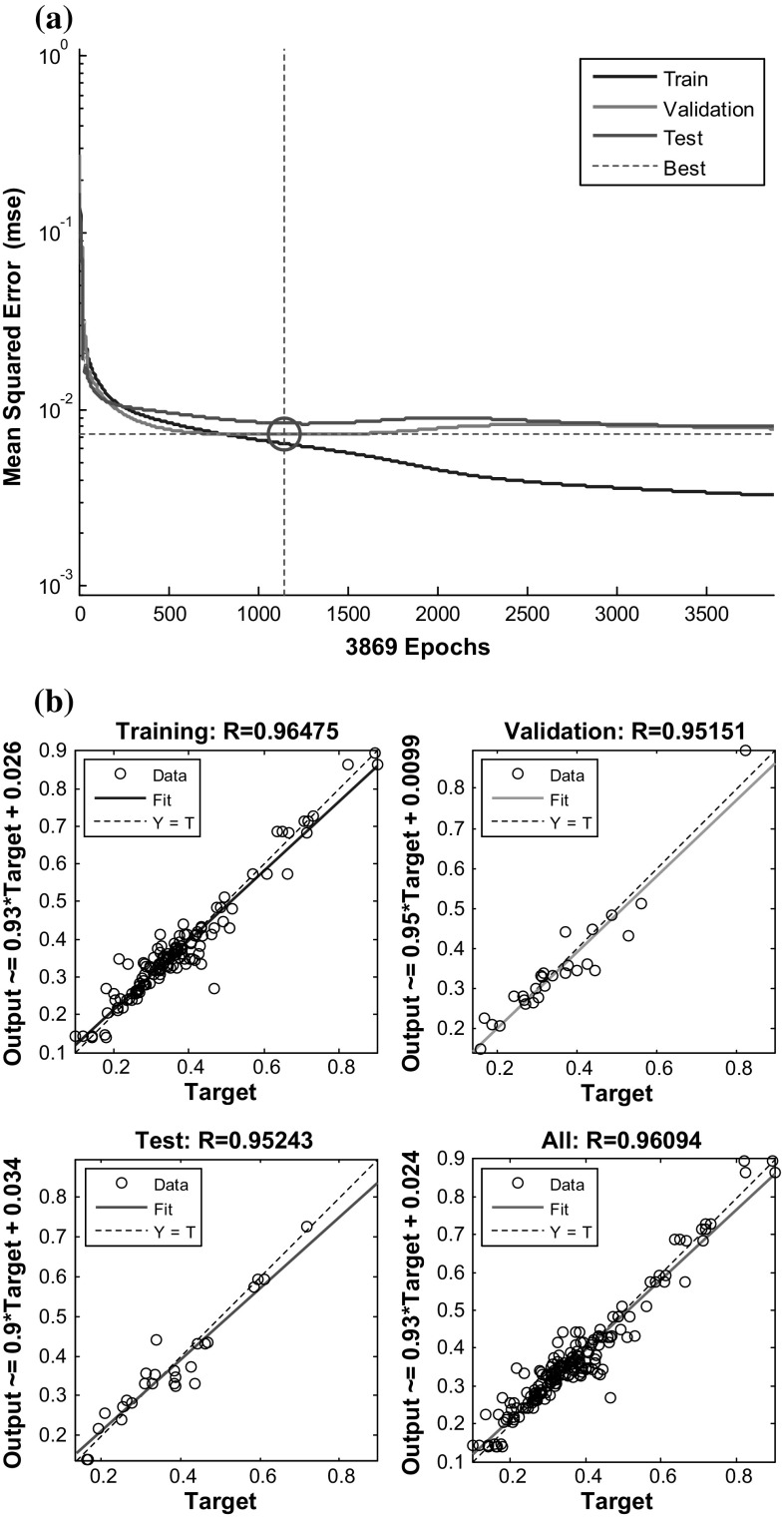
Performance goal of the network, **a** MSE on the validation samples, **b** regression graph for observed and predicted values of the colour change

Next, ANN with the proposed topology was trained and tested. As can be seen from Fig. 10a, the training stopped when the validation error increased after 1147 iterations. The result is sensible because MSE is very small. As can be seen from graph (Fig. 10a), the test and validation errors have similar characteristics. Furthermore, no significant overfitting has followed [58]. Finally, MSE of optimal ANN topology was equal to 0.0072095 (see Fig. 10a).

Figure 10b shows ANN regression plots between outputs and targets samples. The *R* values in each case are greater than 95%. Therefore, the fit is reasonably good for all data sets. Additionally, MAE and *R* for colour change in rehydrated apple cubes were estimated as 0.0259 and 0.96475 for training, 0.0399 and 0.95243 for testing and 0.0264 and 0.95151 for validation data sets. Therefore, the topology with 3 inputs, 13 neurons with 1 hidden layer and 1 output (3–13–1) was applied for predicting the colour change in rehydrated apple cubes.

Comparing the results of the simulation using AG and results from Table 2 (ID. 27), it can be seen that MSE (=0.0072) of optimised ANN topology is smaller compared to the errors values presented in Table 2 (MSE = 0.0074). Moreover, both of the numbers of hidden neurons and epochs are less in case of ANN optimised by genetic algorithm.

### Validation model results

**Fig. 11:**
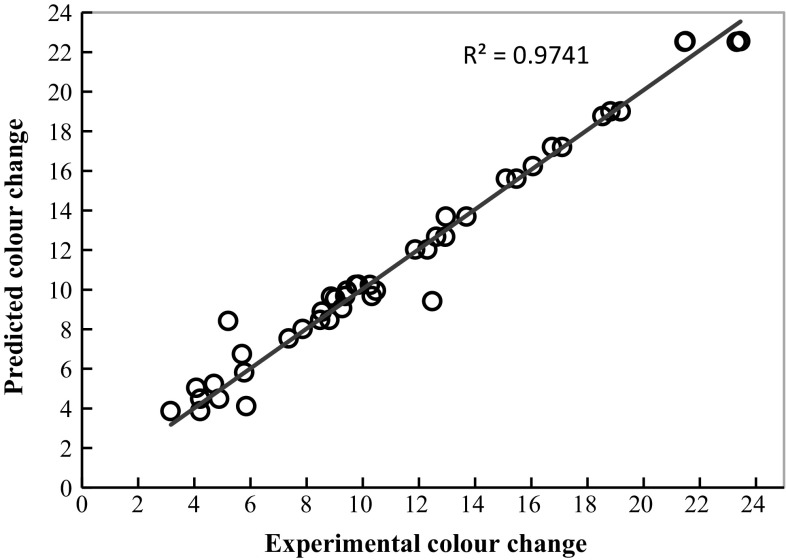
Comparison of predicted and desired output values using optimal neural network topology

According to the authors [32–34], the trained neural network must have a high predictability for new data. Therefore, 40 data sets of colour change obtained from the new experimental run (drying air temperature = 55 °C, drying air velocity = 0.52 m/s, rehydration temperature = 35 °C and rehydration time = 35 min) were used for the verification of the developed model. The regression result of the testing model with new samples is shown in Fig. 11. It can be seen that the genetic algorithm has been successfully applied to optimise of neural network topology. Moreover, the optimised ANN topology was efficient for predicting colour change in the rehydrated apple cubes. This system can also be used for optimising the topology of a neural network which describes the other engineering problems.

## Conclusions

The following conclusions can be drawn from the investigations conducted in this work:

1. An efficient hybrid intelligent approach was proposed to find the optimal topology of neural networks.
2. The optimal ANN topology was more precise for predicting colour change in the rehydrated apple cubes with a low mean square error (0.0072095) and a high regression coefficient (0.96).
3. The optimum neural model had minimum when the number of hidden neurons, learning rate, momentum constant, number of epochs and number of training runs were equal to 13, 0.33, 0.89, 3869 and 3, respectively.
4. The results of the testing model on new trials showed excellent agreement between the actual and predicted data with a coefficient of determination equal to 0.97.
5. This optimisation method significantly reduces the number of experiments comparing with more expansive learning methods.
